# 

**Published:** 1921-08-27

**Authors:** 


August 27, 1921] [Price Twopence

### The Hospital

The Workers' Newspaper of Administrative Medicine and Institutional Life, Administration, National Insurance and Health.

### CONTENTS

**The Medical Profession and the Public Well-Being** 359

**A New Career for Medical Men** ... ... ... 360

**Research and the Medical Student** .. ... ... 361

**Medical Training and Practice in India** ... ... 362

The Military and Civil Services.

**The Institutional Library** .. ... ... ... 363

Diseases of the Ear, Nose, and Throat in Childhood—Materia Medica and Pharmacy—Tuberculosis: its Prevention and Home Treatment.

**Notes of the Week** ... ... ... ... ... 364

Free-will Offerings or Patients' Payments?—Politics and the Irish Hospitals—The Radcliffe Infirmary's Example—The Endowment of Mental Research—Doctors' Fees and Patients' Payments—The Dockyard Fix at Portsmouth—Serious Position of the C.O.S.—Standard Cod-Liver Oil—The Finance of Hospital Magazines—A Dairyman's Conscience.

**The Problem of the Insane** ... ... ... 366

III.—Inside the Asylum.

**The Matrons' and Sisters' Department** ... ... 367

Nursing Under the Ministry of Health.

**Parliament and the Professions** ... ... ... 367

**The Medical Curriculum** ... ... ... ... 368

Qualification and Registration—The English Conjoint Course—The Fellowship of the Royal College of Surgeons—London University Course—Oxford University Course—Cambridge University Course—Higher Diplomas.

**The Medical Student and his Work** ... ... 371

The Medical Schools of the United Kingdom.

**Medical Schools in the Provinces** ... ... ... 374

**Medical Schools in Scotland** ... ... ... ... 375

**Medical Schools in Ireland** ... ... ... ... 376

**Navy, Army, Air and Colonial Services** ... ... 377

**Graduate Education and Institutions** ... ... 378

**Special Hospitals**... ... ... ... ... ... 379

